# Sticky steps and the gender gap: how thoughtful practices could help keep caregivers in science

**DOI:** 10.1098/rspb.2022.1837

**Published:** 2022-11-30

**Authors:** Stephanie Meirmans, Dunja K. Lamatsch, Maurine Neiman

**Affiliations:** ^1^ Department of Ethics, Law and Humanities, Amsterdam UMC, Amsterdam Medical Center, University of Amsterdam, Meibergdreef 9, 1105 AZ Amsterdam, The Netherlands; ^2^ Research Department for Limnology, University of Innsbruck, Mondsee, Mondseestraße 9, 5310 Mondsee, Austria; ^3^ Department of Biology and Department of Gender, Women's, and Sexuality Studies, The University of Iowa, Iowa City, IA 52242, USA

**Keywords:** funding, caregiving, gender, leaky pipeline, Matilda effect

## Abstract

Many fewer women than men hold senior academic positions, a widely recognized and increasing problem. Our goal is to identify effective and feasible solutions. We begin by providing an in-depth assessment of the drivers of this gender inequity. In our synthesis of existing data, we provide many lines of evidence highlighting caregiving as a primary main factor. This is not a ‘new’ insight per se, but a point worth repeating that we back up by a strong and synthetic body of recent data. We also believe that our analysis provides a step forward in tackling a complex issue. We then develop a more detailed understanding of the challenges academic caregivers face and discuss whether and why it is important to keep caregivers in science. We find that the attrition due to caregiving should not be seen as a factor but rather as a process with multiple ‘sticky steps’ that eventually drive caregivers out of science—which, as we argue, is partly also good news. Indeed, it is here that we believe actions could be taken that would have a real impact: for example, one could effectively increase and expand upon current funding practices that focus on caregiver career advancement.

## 1. Introduction

Across the sciences, women are markedly underrepresented in senior-level academic positions and in roles such as editorial board membership [1, p. 116; 2, p. 215ff]. The problem is expected to persist for another century in some locations and for some disciplines [3] (see also [4] for a general overview). This persistent gap is surprising given that the total number of women in science has increased markedly in many disciplines in recent years. There is also a near-closure of the gender gap in many student and PhD populations. The latter suggests that a distinct loss of women in the transition from junior to senior career levels is responsible for the gender gap that still characterizes senior-level positions (e.g. [5,6]).

Immediate action to repair this so-called ‘leaky pipeline’ is a frequent message (e.g. [3,7]), suggesting a wide range of actions. Most policy-based efforts have focused on mentoring schemes and leadership training but have shown little efficacy to date [8]. Indeed, attrition of senior female scientists within the last 30 years remains a challenge and has even increased in some situations, leading Lawton Smith et al. [9] in their recent book on gender and science to claim a ‘broken pipeline’.

Other recent scholars have argued against the concept of a leaky pipeline. Some argue that a different metaphor is more apt, such as ‘chutes and ladders’ [10]. Another perspective is that the concept of a pipeline might be problematic in providing justification to continue filling vacant leadership positions with the typical subjects—white men.

How can we reduce the gender gap at senior academic levels? In this paper, we address this issue in a novel synthesis framework by first asking (i) what are the most prominent factors driving the leak? And because these factors seem to be associated with women's role as caregivers, we also ask the following questions. (ii) Are women indeed the only victims of caregiving-associated challenges? (iii) Why is it necessary to keep caregivers in science? (iv) Why is the leaky pipeline getting worse rather than better, despite recent actions? (v) How can we effectively improve the situation? (vi) How can we use funding practices to better support academic caregivers?

## 2. What are the most prominent factors driving the leak of women from academia?

It is widely recognized that the human female biological reproductive prime coincides with typical PhD and postdoc years. Some people consciously postpone childbearing or childrearing until after tenure, but with the tradeoff that the reduction of fertility with age can translate into challenges with achieving a healthy pregnancy and life fulfilment [11,12]. After reviewing all then-available evidence concerning the academic gender gap, Ceci & Williams [13] concluded that fertility choices and work–home balance are the main forces underlying the gender gap across all scientific disciplines in recent years. Similarly, a recent European consortium study that explored the causes of gender bias in science across six European countries at multiple scales indicated that caregiving, including but not limited to parenting, during the vulnerable postdoc and tenure-track period is the major barrier towards the retention of women in an often-hypercompetitive environment [14].

In addition, Cech & Blair-Loy [15] show that in the USA, nearly 50% of women and nearly 25% of men leave a full-time job in STEM science after becoming parents. Using bibliometric data of 1.5 million gender-identified authors across 83 countries and 13 disciplines, Huang *et al*. [16] find that the article-productivity gender gap has been growing over the last 40 years (see their electronic supplementary material, figure S10d). Huang *et al*. also find that this gap is largely explained by higher dropout rates of women and their reduced career length relative to men. When adjusting for these components, article productivity becomes much more similar across genders.

Using survey data from the USA and Canada, Morgan *et al*. [17] find that parenthood is the primary source of the gender gap in article productivity in these two countries. They also show that this gap has been decreasing in recent years as a consequence of increasing availability of parental leave and appropriate childcare. Zheng *et al*. [18] used surveys in combination with biobliometric data to discover that gender gaps in academic achievement are, in fact, ‘parenthood gender gaps’. They also show that mothers are more negatively affected because they experience a higher level of work-family conflict and lower levels of support from their partner. This phenomenon contributes to the gender gap in academic achievement. Finally, the COVID-19 pandemic serves as a natural experiment highlighting the impact of caregiving on scientists and their careers, in particular if supportive structures, such as day-care/kindergarten and school, are unavailable [19–22].

Discrimination and bias against mothers in the workforce are collectively often called the ‘motherhood penalty’ [23]. The wage motherhood penalty does vary across countries, however, depending on support for childcare as well as cultural factors [24]. This variation hints at how we might make improvements. For example, the motherhood wage penalty in Scandinavian countries is relatively low due to relatively high cultural and institutional support. In other countries (e.g. Austria) the penalty is relatively high [24].

Together, these studies provide multiple lines of evidence that combining a time-intensive career in science with family life and caregiving is a main driver of the gender gap in senior and leadership science positions. It also seems that thoughtful and impactful attention to this issue could counteract the leakage of women from junior to senior academic positions and help close the gender gap.

## 3. Are women indeed the only victims of caregiving-associated challenges?

Many recent reports and papers highlight that child-related career challenges are more broadly linked to a bias against caregivers per se rather than women (see several examples in [15,25,26]). Thus, it is important to note that fathers who are primary or co-caregivers can be equally affected (see also [17,27]). Scientists caring for elderly, dependent, or ill family or friends also often face similar biases or barriers, meaning these other caregivers are in need of supportive structures as well (examples in [25,26]).

That said, it is important to note that structural and emotional investment in care, regardless of gender, also plays an important role. Derrick *et al*. [28] used a survey including over 11 000 scientists in connection with bibliometric data on scientific productivity to investigate how parental investment affects scientific productivity. These authors distinguished between ‘lead parents’, performing the major share in caregiving; ‘dual parents’, shared caregiving responsibilities with a partner; and ‘satellite parent’, contributing the minor share in caregiving. They found that lead-parenting men suffer from a similar negative productivity effect as do lead-parenting women. This effect is diluted by the fact that the percentage of men in academia who are lead persons in parenting engagements is nearly eight times lower (4%) than for women (31%). Derrick and colleagues also detected a subjective bias regarding the shared responsibilities in dual-parenting couples: even if couples indicated that they shared the care work equally, women actually took on a larger share of the parental responsibilities. In parallel, satellite-parenting women also took a larger share of the parenting work than did satellite-parenting men. This phenomenon might explain reports like that of Dalum *et al*. [29], using a dataset representing all STEM researchers in Denmark, who found that Danish mothers are 24% more negatively affected in their academic article productivity on an annual basis after their first childbirth relative to fathers. It is also relevant to note that Elliott [30] found that women also take a larger share in caring for their elderly parents than do men. Indeed, increasingly many women (and men) care both for children and their parents—recently described as the ‘sandwich generation’.

It is important to also recognize the distinct potential for intersectionality in this context: that challenges connected to caregiving might often be amplified by other challenges facing certain underrepresented groups. For example, a non-white caregiver might simultaneously experience caregiving-associated challenges with fieldwork as well as racism while in the field (see e.g. [31]). These amplified effects linked to intersectionality are generally well described (see e.g. [32]) and probably also often play a role in connection with caregiving. While we appreciate that intersectionality is a very important and related issue, its complexity and scope is too large to provide it the attention it requires in this paper along with our main focus on caregiving and gender.

We are also aware that some scientists will not identify as genders captured in the ‘woman/man’ binary. That these other gender identities have only recently become part of the popular and scientific lexicon means that there do not yet exist many datasets that include these gender distinctions. We recognize that our inability to include gender identities outside of man/woman poses a limitation, but we hope and believe that future treatments of issues related to caregiving will be able to provide comprehensive analyses of a broader spectrum of gender identity.

## 4. Why is it necessary to keep caregivers in science?

Why bother keeping caregivers in academia? From a diversity, equity and inclusion perspective, science should fairly represent all of humanity. We also know that diverse teams perform better under the right conditions, including elements like inclusiveness, true integration and flat hierarchies [33,34]. Finally, caregivers have a lot to offer to science: caregiving enhances skills critical to research, teaching, appreciative communication and mentoring such as responsibility, reflexivity, practicality, organizational talent, empathy, perspective and social engagement. The ability to adopt the perspective of others could also help ease challenges associated with inter- and transdisciplinary research and diverse teams, as well as bridge the gap between science and the public.

The inclusion of researchers with a good work–life balance could also be a healthy antidote to the current ‘greedy model’ of academic work defined as excellence in all work-related aspects (see [35]), that has made unhealthy work styles the norm at most universities today [36]. Indeed, several case studies indicate that caregiving could foster better work–life balance, including increasing scientific quality through more extensive ‘thinking time’, facilitating team building and collaboration, and opening doors to public engagement [37,38]. Altogether, science as an enterprise might thus be better off with a healthy community that includes caregivers.

## 5. Why is the leaky pipeline getting worse rather than better, despite recent actions?

The gender gap has increased when compared to 30 years ago [9,16]. We suggest that one mechanism that could be driving this phenomenon is intense competition among scientists [36] occurring simultaneously with substantial caregiving duties (see e.g. [14]). Notably, not only the *degree* of competition has increased, but also the overall *duration* of competition: for example, the time period between Ph.D. and permanent employment becomes ever longer. Dubois-Shaik & Fusulier [14] found that it is within these prolonged PhD/postdoctoral phases of the career that female scientists seem to be particularly vulnerable. Thus, it is nowadays not a ‘sticky floor’ that keeps caregivers, the majority of which are women, at the bottom of work hierarchies, but rather a row of multiple ‘sticky steps’ [39,40] (see also [7]).

For example, consider the often-made observation that caregivers frequently seem to voluntarily make the choice to leave science (see e.g. [5,13]). Caregivers also often choose part-time positions, which offer obvious advantages for caregiving but do not typically translate into a high-profile academic career. Indeed, women are nowadays more likely to work part-time than men in a majority of European countries ([1, p. 97]).

We believe that these career choices are not entirely 'voluntary' and that such representations of a binary career pathway are an oversimplification that miss critical nuance. Instead, the accumulation of small career-related decisions that may seem relatively minor at each step later often result in caregivers opting partially or fully out of science. Decisions often represent practical choices within family structures; for example, a father, often older than a mother, already has a permanent career, so it becomes natural for the mother to take a presumed temporary break and/or a reduced workload. There is also a common practice of taking an extended postdoctoral position abroad. However, these postdocs abroad are often spent away from other potential carers such as close family that could act as ‘alloparents’ (*sensu* [41]). It could even mean physical extended separation from the partner/father, who might have a position elsewhere. All of these issues pose substantial challenges with respect to the retention of many new parents in science.

Funding represents another major issue for caregivers. Traditional academic success in science requires acquiring substantial grant funding, and often even in the period before permanent employment. However, competition for grants is often fierce. A successful application also depends heavily on publication record and other types of scientific ‘credit’, such as awards or invited talks, that are viewed as good predictors of future success. This link between funding success and metrics of productivity means that caregivers—typically compared to non-caregiving scientists that often devote all of their available time to science—might often fall short in grant competitions. Windows of opportunity for particular funding opportunities (e.g. within a certain number of years after receiving a PhD) can close quickly, leaving no time for delay. Indeed, observing attrition of caregiving scientists around them, many new caregivers see funding acquisition as a lost battle from the start and opt out of science immediately after becoming a parent (see e.g. [5]).

Because funding plays an increasingly central role for career advancement, current funding practices also often inhibit rather than elevate women and caregivers. A major challenge in science funding is posed by the so-called ‘Matthew effect’, where those scientists that already have garnered traditional metrics of productivity or success are more likely to receive additional such credits (after [42]). The implications of the Matthew effect also mean that scientists with few initial successes will find it relatively difficult to achieve successes in the future. This cascading consequence of the Matthew effect has also been called the ‘Matilda effect’ because this phenomenon is thought to be especially likely to affect the careers of women [43]. In the extreme case, and as documented on multiple occasions historically, women collaborators do not receive any recognition for the work they have done, with scientific success attributed instead to their male colleagues or husbands [43]. In addition, recent work in economics suggests that women, in contrast with men, are less likely to receive tenure when their work is co-authored [44].

Do we indeed see this Matilda effect in science funding? Rissler *et al*. [45] reviewed the evidence regarding the outcomes of research grant applications submitted by women. This analysis revealed that, across multiple countries, women and men who apply have equal chances of success, though this outcome might differ for e.g. ERC grants [46]. Rissler *et al*. [45], however, also found that fewer women submit applications than men, translating into fewer research grants to women. With specific respect to the USA, they argued that lower application rates to NSF research grants for women versus men could be a consequence of the typically higher teaching and service loads for women. Steinþórsdóttir *et al*. [47], who have very similar findings for funding in Iceland, call this type of work ‘academic housework’. More time in teaching and service means less time for research, with the consequence that some women might not be, or perceive themselves not to be, competitive enough to apply for research grants. The implications are that the Matilda effect does seem to influence the rate of application by women for research grant support.

One could argue that women simply should submit applications more often. This remedy is unfortunately too simplistic, failing to recognize the real underlying problems (see also [47]). Also, as Fritch *et al*. [48] have recently shown for Ireland, certain eligibility restrictions may simply prevent women from applying. Such restrictions could in practice only be counteracted by actively changing those restrictions, such as the number of senior-authored papers required to apply. That said, it is worthwhile noting that at least at some of the highest levels of competition, namely for ERC grants, women indeed have markedly lower success rates per application than men, indicating an ‘intangible glass ceiling’ for those types of competitive research grants [46].

All of these issues are compounded not only by the frequent need to compete for funding, but also by caregiving of multiple children or other dependants as time passes. Indeed, challenges linked to multiple dependants seem to be only rarely addressed in the literature (for an exception see [27]). It is also a common misconception that children do not require dedicated focus once they attain school age. In other words, care of multiple/older children or dependants needs to be considered and taken into account. A relevant example comes from Ecklund & Lincoln [27], who found that scientists—mothers, and in particular fathers—often have fewer children than desired as a consequence of career considerations, leading to overall reduced life satisfaction.

Altogether, we believe that caregivers typically face such a multitude of such challenges that this leads to a career ladder full of ‘sticky steps’ [39,40] (see also [7]), with each step getting more difficult to reach as a caregiver.

## 6. How can we effectively improve the situation?

As described above, a main driver of the loss of caregivers from academic science is that traditional academic structures often work against them. The typical way to respond to these challenges is to help caregivers to ‘achieve it all’ in order to align them more with non-caregivers, via e.g. offering free or subsidized full-time childcare.

But there may be other options: Ceci & Williams [13] advocated that ‘the linear career path of the modal male scientist’ should be abandoned as a standard in science and that universities and funders should experiment with alternate life courses. Inspiration for these options could come from personal accounts that focus on how to strike a balance between career and family (see e.g. Royal Society reports *Mothers in science* [49] and *Parent carer scientist* [25]; series of papers in the journal *Evolutionary Applications* [50]; series of papers in *eLife* ‘Scientist and parent’ [26]). Indeed, what these reports highlight is that there are a variety of career options that can translate into fruitful combinations of caregiving and science. It is relevant to note that these reports include multiple instances of ultimately successful female scientists who have taken extensive maternal leaves in the past and subsequently returned to science. In general, more flexibility and a more explicit consideration of alternate life course options should help caregivers stay in science.

## 7. How can we use funding practices to better support academic caregivers?

In our opinion, one potentially powerful way to increase retention of caregivers in science could be via more fully expanding existing and new ways to counteract the Matilda effect that influences funding decisions (see also [13,14]). In addition, one could implement strategies that actively help caregivers to move up the sticky career ladder. Below, we highlight some of the already existing best funding practices that take into account the trade-offs that working mothers and other caregivers face and support these individuals in their choices where needed (see also table 1 for an overview; see electronic supplementary material for an extended version of sections I–IV, including additional information and examples). To this table, we have also added some new ideas for how funding schemes could support caregivers.

**Table 1.** Overview of existing and new funding practices that could help caregivers.

remove or adapt funding criteria that disfavour caregivers	reduce the emphasis on leadership qualities/ applicant CV in some grant evaluations
	eligibility I: lowering eligibility requirements of productivity/excellence
	eligibility II: provide reasonable windows of opportunity via extensions to caregivers
	eligibility III: introducing alternative requirements for ‘time since PhD’
	provide more flexibility in mobility grants
	deadlines: make family-conscious deadline decisions
raise awareness for grant reviewers and panel members about the challenges caregivers face	educate panels and reviewers on what it means to juggle a career and caregiving
	introducing a grant section that explicitly addresses caring-related career gaps
	implement measurements to ease grant submission process for caregivers
provide extra money and time to caregivers via grants	paid day-care and extra expenditures
	extend grant length
	provide extra funding to hire a helper
	special conference funding
provide special grants focused on caregiver research programs	provide more and different career gap grants
	parent–tandem grants
	special professorship grants

### (a) Remove or adapt funding criteria that disfavour caregivers

#### (i) Reduce the emphasis on leadership qualities/ applicant CV in some grant evaluations

Traditionally, funding agencies select proposals via a focus on research project quality. However, funding schemes also often evaluate proposals primarily via traditional metrics of applicant quality (e.g. leadership qualities and CV). We here argue that such a focus might generate a bias against caregivers via the Matilda effect described above. Caregivers might thus fare better if grant evaluation in such schemes focused more on project versus candidate quality.

#### (ii) Eligibility I: lowering eligibility requirements of productivity/excellence

In general, relaxing productivity and excellence eligibility requirements for grants and other fellowships could increase applications from caregivers, as caregivers likely had less time and fewer opportunities to acquire career-related metrics reflecting productivity and excellence.

#### (iii) Eligibility II: provide reasonable windows of opportunity via extensions to caregivers

Restricted ‘windows of opportunity’ within academic careers present a major challenge for scientists with caregiving responsibilities (see also [14,51]). For many talent grants, there are restrictions connected to eligibility that are based on chronological or academic age, the latter defined by time since PhD, and those windows quickly close. Therefore, extensions of eligibility periods concerning such windows of opportunity that are in line with the actual caregiving provided can make all the difference when it comes to caregivers and their scientific career.

#### (iv) Eligibility III: introducing alternative requirements for ‘time since PhD’

As described above, windows of opportunity in academia quickly close, and missing an early window because of, say, motherhood, can also translate into more missed opportunities later. There is thus a problem of cascading consequences of early missed opportunities (see e.g. [52]). Other structures could be conceived that would be less likely to disadvantage caregivers.

#### (v) Provide more flexibility in mobility grants

In Europe, a prominent postdoctoral funding opportunity is provided via the Marie Skłodowska Curie program. This program requires mobility of the postdoc, which typically involves moving to another European country. However, such mobility requirements can also present a real hurdle for parents and other caregivers [14]. This possibility finds support from Derrick *et al*. [28], who showed via a qualitative analysis that mobility requirements present a major burden to parents. We believe that a more careful consideration—and potential relaxation—of mobility requirements would enable caregiving scientists to apply more often to mobility funding schemes, thus being able to partake in the positive effects of mobility at least to some extent.

#### (vi) Deadlines: make family-conscious deadline decisions

Deadlines for grant applications often fall into the end of a vacation or holiday period. This timing might come with administrative or logistic benefits for some. Nevertheless, the timing of such deadlines is likely to be difficult for caregiving researchers applying for such types of grants for the simple reason that caregiving researchers are very often primarily in a caregiving role during holidays and vacations. We therefore suggest that moving deadlines to other times of the year, and/or removing deadlines altogether could be helpful for caregiving scientists. A similar issue applies to grant reviews that fall in the vacation period. This in turn might mean that those researchers who give care to their children and other dependants will be less able to take on such prestige-building tasks.

### (b) Raise awareness for grant reviewers and panel members about the challenges caregivers face

#### (i) Educate panels and reviewers on what it means to juggle a career and caregiving

While we acknowledge that many scientists are aware of the challenges of combining caregiving and scientific careers, room for improvement remains. We suggest that similar to the widely used and successfully implemented implicit bias training, panels and reviewers could receive training focused on challenges posed by caregiving as well as messaging that these challenges do not mean a scientist's work is necessarily of lower quality or less influential. Important in this setting might be new, more holistic ways to evaluate researchers.

#### (ii) Introducing a grant section that explicitly addresses caring-related career gaps

In Australia, career choices can be justified in a so-called Research Opportunity and Performance Evidence (ROPE) statement. In this ROPE statement, researchers can, for example, note research interruptions due to caring and parental responsibilities. Some Austrian FWF funding schemes include an academic CV section within the application that allows for elucidation of parental leaves and part-time work linked to parenting. Such explanations should enable grant reviewers to better and more fairly evaluate the applicants (e.g. by measuring efficiency rather than mere quantity). Indeed, we believe that an opportunity for explanation of career gaps or periods of relatively low productivity should be explicitly required for all major grant applications.

#### (iii) Implement measurements to ease grant submission process for caregivers

The Dutch funder NWO recently introduced a ‘compensation scheme’ for scientists that face challenges during the grant evaluation process linked to the birth of a child, the arrival of an adoptive or foster child, or other forms of ‘force majeur’ put upon a caregiver. This could, for example, mean that writing a rebuttal or taking part in an interview at a specific moment might be a difficult task for a caregiver. In this case, scientists can apply for a later date for an interview, can interview via a remote platform like Zoom or Skype or can receive an extension for the rebuttal deadline.

### (c) Provide extra money and time to caregivers via grants

#### (i) Paid day-care and extra expenditures

In some types of grants (e.g. in Switzerland and Austria), grant recipients can apply for extra funding to support childcare and other expenses related to childrearing. We suggest that such support for caregiving and related expenses should become part of standard granting schemes. In addition, applications should be structured in a way that these funds are easy to request. One could for example include a specific category in grant budgets that is explicitly set aside for caregiving so researchers do not feel that they are asking for a favour or special treatment when requesting funds for caregiving. In addition, it is important to provide the extra money for caregivers via a specific funding stream that is separate from the total budget for any given funding purpose. This funding structure is important in order to avert a situation where reviewers or panel members exhibit bias against caregivers because their selection might mean less money for others in the funding round. The same consideration applies to further arguments for providing extra money for caregivers laid out below.

#### (ii) Extend grant length

Ceci & Williams [13] generally recommend extending grant length when caregiving responsibilities are substantial. Such types of extensions could be provided to new parents along with caregivers of older children or other dependants.

#### (iii) Provide extra funding to hire a helper

The US National Institutes of Health as well as the US National Science Foundation provide funding schemes that allow a postdoctoral researcher with an NIH grant who is also a parent to hire a laboratory assistant. One could also imagine expanding this form of support to include funding that enables use of other forms of assistance, e.g. administrative duties, statistical advice or paper editing, that could make a qualitative difference for early-career scientist-caregivers.

#### (iv) Special conference funding

For caregivers, attending a conference abroad can be quite a hurdle. Some societies, e.g. ESEB, have started to offer funding for caregivers that can be used to support childcare at home during the meeting or at the conference itself. These two options are important for at least two reasons. First, caregiving structures at conference sites often do not cater to older children. It can also be very difficult to leave an anxious child within day-care structures that are entirely new for them, often including a language foreign to the child, and/or involve non-trained personnel. For breastfeeding children, barriers to feeding can arise if the day-care is not on-site. For single mothers or fathers, it can be difficult to impossible to either travel alone with children or to leave children at home if there is no overnight care available. Funding support to provide care to other types of dependants as well (e.g. an elderly parent) are also important.

Another non-mutually exclusive solution for universities is to provide grants to caregiving academics that support caregiving needs (e.g. an electric breast pump, a plane ticket for a caregiving partner or babysitting), in order to enable them to engage in professional activities.

### (d) Provide special grants focused on caregiver research programs

#### (i) Provide more and different career gap grants

Ceci & Williams [13] suggested providing grants after leaves of absence for caregivers. There already exist some career gap-focused grants from the UK and from the EU. These types of grants might be particularly crucial for caregivers that have taken extended time off. One challenge posed by grant schemes like the re-entry Marie Skłodowska Curie grants, however, is the requirement imposed on the scientist to move countries. We therefore suggest moving such career gap grants into a different section of the European funding program—a section that does not require such mobility. We also suggest that career gap funding programs should also be taken up by other national funding agencies.

#### (ii) Parent–tandem grants

We propose that a shared grant scheme might provide a major advance for academic couples with children and even contribute towards a solution for the formidable ‘two-body problem’ of academic partners [53,54]. One of the main challenges for academic couples is to find a position in the same city or even country. This problem can be exacerbated for field biologists because they often study sites or organisms located in very remote locales or are situated in remote research institutes. Such grants could provide funding for couples that enables them to work in the same location and share caregiving responsibilities. One could also consider preferential employment for a partner to a scientist whose work demands substantial time in a remote location.

#### (iii) Special professorship grants

There is a growing set of institutions and funding agencies that support programs aimed at increasing representation of women at senior academic levels. For example, at Groningen University, the Netherlands, women can apply for prestigious Rosalind Franklin fellowships that provide tenure-track positions and a generous start-up package. In Austria, there are two grant programs available on an annual cycle, Herta Firnberg and Lise Meitner, that are aimed at helping women attain a level of qualification that allows them to apply for professorial positions within Austria or abroad. In addition, these grant applications that could not be funded by FWF due to financial shortage but are rated above average will automatically be transferred to another funding body to enhance chances for women. We suggest that it would also be helpful to include some or all of the above-mentioned specific aspects to mind caregivers in such schemes as well.

## 8. Conclusion

In this paper, we provided a novel synthesis framework for how to understand and address a seemingly complex and heretofore intractable problem: the gender gap in science. We show that an accumulating body of qualitative and quantitative evidence suggests that this problem is driven by one main factor: caregiving, which is predominantly provided by women. Recognizing the simplicity within complexity is important to leverage policy changes that matter. In an effort to provide actionable and feasible suggestions for improvement, we also provided a more detailed account of what is happening—which is indeed not a ‘factor’ but a process. We used the metaphor of ‘sticky steps’ to highlight that there is typically an incremental process of attrition of caregivers over the course of academic life.

The implications are that helping caregivers should decrease the gender gap. In particular, one should create more flexibility in career advancement. Personal ‘case studies’ investigations [14] show that the careers of mothers in precarious times, such as e.g. postdoctoral periods, can particularly benefit from a combination of structured and personal support and understanding and reduced focus on traditional metrics of excellence (see also [35]). For example, there should be more structures that facilitate re-entrance into science at later career points, with the ultimate goal of keeping caregivers who want senior-level scientific careers in academia. With these points in mind, we also reviewed existing funding practices that could be beneficial to caregivers as well as made suggestions for tweaks and new ideas.

We would also like to note that many of the strategies that we suggest in our paper might also help the academic community in general and could contribute to the diversity of research environments. In particular, the acceptance of a wide variety of career paths, more respect for private time, flexible or extended deadlines, and broadening of eligibility limits for grant applications could help improve equity, inclusion and diversity with respect to the scientific community.

## Data Availability

Additional information is provided in the electronic supplementary material [55].
